# An integrated chemical and biomarker approach using *Ucides cordatus* (Crustacea; Decapoda) to assess trace element contamination and its biological impacts across two Amazonian estuaries

**DOI:** 10.1007/s10661-026-16077-4

**Published:** 2026-10-21

**Authors:** Luciana Barros Oliveira, Lina Clara Gayoso e Almendra Ibiapina Moreno, Suelen Rosana Sampaio de Oliveira, Laércio dos Anjos Benjamin, Débora Batista Pinheiro-Sousa, Gerson dos Santos Protázio, Alana Lislea de Sousa, Thales Passos Andrade, Eliane Braga Ribeiro, Jonatas da Silva Castro, Gustavo Henrique Rodrigues Vale de Macedo, Luan Jonatas da Silva Ferreira, Verônica Maria Oliveira, Débora Martins Silva Santos, Sandra Fernanda Loureiro de Castro Nunes, Raimunda Nonata Fortes Carvalho Neta

**Affiliations:** 1 Postgraduate Program in Biodiversity and Biotechnology (REDE BIONORTE), State University of Maranhão, São Luís, MA CEP 65055-310 Brazil; 2 Postgraduate Program in Ecology and Biodiversity Conservation, State University of Maranhão, Paulo VI Campus, São Luís, MA CEP 65055-970 Brazil; 3 Department of Veterinary Medicine, Federal University of Viçosa, Viçosa, MG CEP 36570-000 Brazil; 4 Coordination of the Environmental Engineering Program, Federal University of Maranhão, Balsas, MA CEP 65800-000 Brazil; 5 Postgraduate Program in Agriculture and Environment, State University of Maranhão, Paulo VI Campus, São Luís, MA CEP 65055-970 Brazil; 6 Department of Fisheries Engineering, Federal University of Maranhão, Pinheiro, MA CEP 65200-000 Brazil; 7 Departmen of Biology, State University of Maranhão, Paulo VI Campus, São Luís, MA CEP 65055-970 Brazil

**Keywords:** Crustacean, Port areas, Biomonitoring, São Marcos Bay, Maranhão

## Abstract

In this study, an integrated chemical and biomarker approach was applied using the mangrove crab *Ucides cordatus* to assess trace element contamination and its biological impacts across two distinct Amazonian estuaries. Crabs were collected from São José Bay (A1—minimally impacted) and São Marcos Bay (A2—impacted by port activities) on the Amazon coast of Brazil. Simultaneously with crab sampling, physicochemical parameters and trace element levels in sediment and water samples were assessed. Biometric measurements included cephalothorax length and width, cheliped propodus length and width, movable finger length and width, and total weight. Two biomarkers, genotoxic responses (micronuclei) and histopathological lesions (gills and liver hepatopancreas), were measured. Abiotic parameters, such as pH, dissolved oxygen, and salinity, were similar between the two study areas (A1 and A2). However, the concentrations of trace elements (Zn, Fe, Al, and Mn) in the water were increased in A2. Biometric data revealed that crabs from A2 were significantly smaller and lighter (*p* < 0.05) than those from A1. Gill lesions (marginal canal deformation, pillar cell rupture, and necrosis) were frequently observed in crabs from both sites, with a higher prevalence in A2. Hepatopancreas histological alterations (e.g., necrosis) were significantly more common in crabs from A2 (*p* < 0.05). Genotoxic responses, such as pycnotic nuclei, were also significantly more frequent in A2 (*p* < 0.05). The results demonstrate that *Ucides cordatus* effectively reflects environmental contamination through measurable histological and genotoxic biomarkers, which is consistent with the chemical evidence of trace element pollution in estuarine environments.

## Introduction

Mangroves are transitional coastal ecosystems that are typically found in tropical and subtropical regions and are strongly influenced by tidal dynamics (Hogarth, 2015; Silva et al., 2024). These environments provide critical resources and ecosystem services and play a vital role in the feeding, sheltering, and reproduction of numerous aquatic species of high ecological, social, and economic importance (Duarte et al., 2020; Mitra, 2020; Ross et al., 2019). However, these habitats have been increasingly impacted by pollution and environmental degradation from both domestic and industrial sources, raising significant concerns regarding the health of mangrove-associated fauna (Aghabozorgi Nafchi & Chamani, 2019; Häder et al., 2020; Salgado et al., 2021; Santos et al., 2019). Organisms inhabiting the mangrove areas of São José Bay and São Marcos Bay (Amazon coast, Brazil) are exposed to a wide array of toxic contaminants associated with sediments, suspended particulate matter, and dietary intake (Righi et al., 2022). Furthermore, benthic animals, such as crabs, are particularly susceptible to the accumulation of these pollutants and suffer from the toxic effects they induce (NOAA 2021). Crabs are more susceptible to exposure and accumulation of contaminants because they live in direct contact with sediment, where metals, organic compounds, and contaminated particles are retained. In addition to direct exposure through contact, these organisms can incorporate contaminants through feeding (Jesus et al., 2021).

The crab *Ucides cordatus* is a semiterrestrial species found exclusively in mangrove ecosystems (Nobre et al., 2022). It plays a vital ecological and economic role along the Brazilian coast, serving as an important source of food and income for communities that rely on its harvesting and trade (Duarte et al., 2017; Jesus et al., 2020a; Pinheiro et al., 2013; Wunderlich et al., 2008). *U. cordatus* is also a useful biomonitor for assessing mangrove environmental quality, as exposure to contaminants can trigger molecular, biochemical, behavioral, and genotoxic changes (Duarte et al., 2016, 2019; Pinheiro et al., 2013). Moreover, it exhibits early responses to trace element exposure (Carvalho-Neta et al., 2014, 2019). In this context, *Ucides cordatus* is a promising species for studying biomarkers of environmental degradation.

Biomarkers reflect the biological responses of organisms to environmental disturbances or contamination, enabling the detection of pollution even at low concentrations (Altenburger et al., 2019). These markers are widely used in biomonitoring programs as early warning tools for the toxic effects of contaminants on organisms (Cajaraville et al., 2000; Martín-Díaz et al., 2004; Solé et al., 2009). Lower-level biological biomarkers (molecular, cellular) show faster responses to mild disturbances, unlike higher-level biomarkers (such as survival and mortality or changes in the population or community). From this perspective, histological and genotoxic biomarkers can be applied to assess organismal exposure to trace elements (Kumari et al., 2017; Negro & Collins, 2017; Obiakor et al., 2014; Sardi et al., 2016; Zhu et al., 2018). The gills and hepatopancreas of crabs are metabolically active tissues that exhibit biological responses that serve as histological biomarkers; namely, the gills are in constant direct contact with pollutants in the water, while the hepatopancreas is the main organ responsible for contaminant biotransformation (Jesus et al., 2021). Additionally, studies using hemolymph have shown that the micronucleus test in these animals can detect chromosomal alterations, breaks, and losses, making it a viable genotoxic indicator (Hayashi, 2016). In this scenario, an integrated chemical and biomarker approach using the mangrove crab *Ucides cordatus* is applied to assess trace element contamination and its biological impacts across two distinct Amazonian bays (Brazil).

## Materials and methods

### Study area

**Fig. 1:**
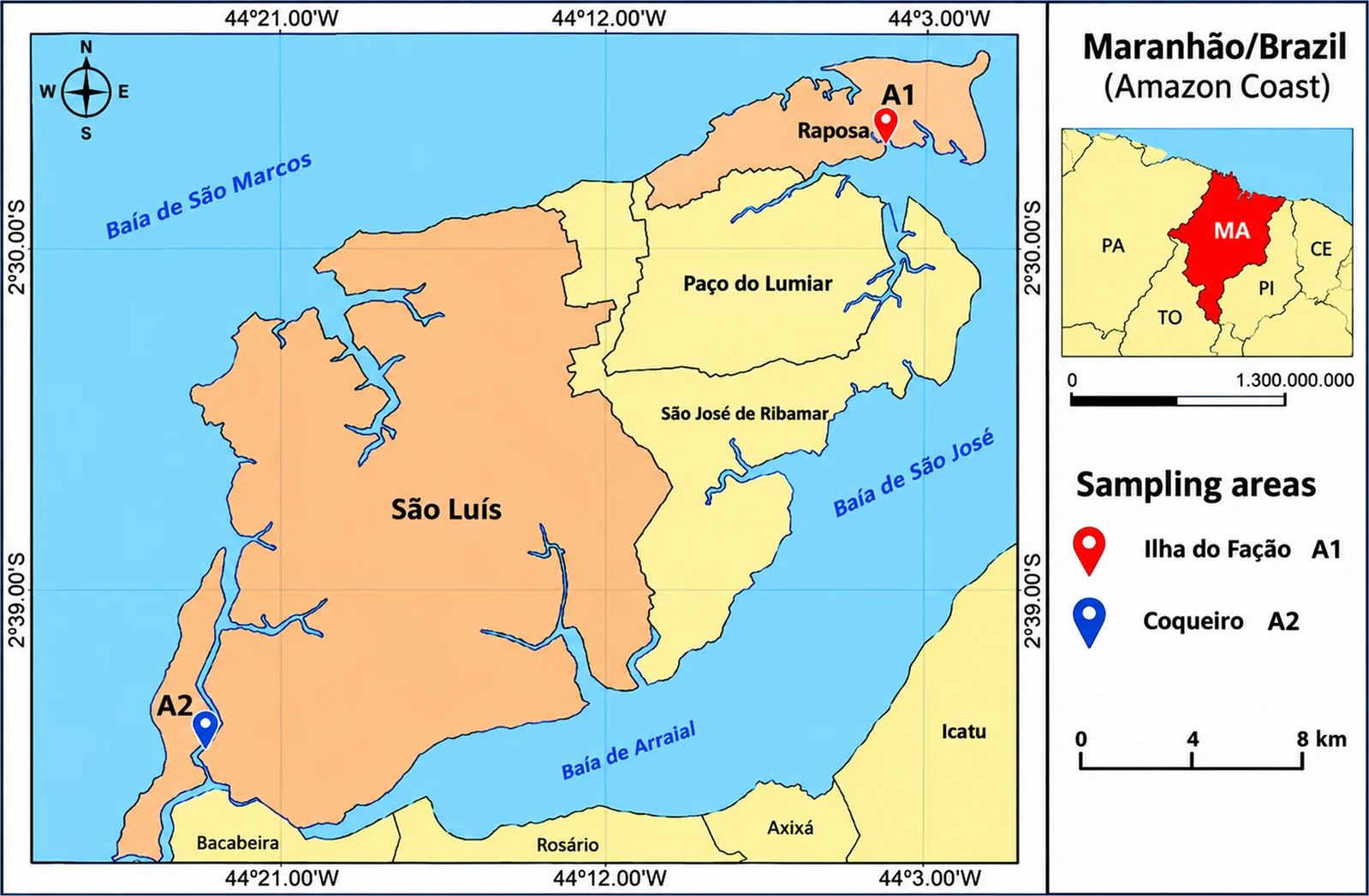
Location of the sampling areas: São José Bay (A1 = Facão Island) and São Marcos Bay (A2 = Coqueiro Community), Maranhão, Brazil

The selected mangrove areas are located along São José Bay and São Marcos Bay on the Amazon coast in the state of Maranhão, Brazil. The first study site is situated on Facão Island (2°26′29.6″S, 44°03′53.5″W), in the municipality of Raposa, within São José Bay (A1 = low-impact area). The second site is located in the Coqueiro Community (2°43′16.6″S, 44°21′39.7″W), in the municipality of São Luís, within São Marcos Bay (A2 = area impacted by port activities). Near this riverside community, there are numerous residences as well as the Ponta da Madeira Port Complex, the Port of Itaqui, and the Alumar/ALCOA Port (EMAP 2021; Jesus et al., 2021). This region experiences continuous cargo ship traffic, with both domestic and international freight operations (Sousa et al., 2013). The sampling sites (A1 and A2) are important to local fishing communities (Fig. 1), which harvest the mangrove crab *U. cordatus* for consumption and trade.

### Physicochemical parameters and determination of trace elements in water

The physicochemical parameters of the water, including pH, dissolved oxygen (mg/L), temperature (°C), and salinity (ppm), at each sampling site were measured twice during the rainy season and twice during the dry season at each sampling location in 2021. Measurements were taken at 12:00 PM (local time for each area) using an HI 9829 multiparameter meter (Hanna Instruments, Woonsocket, RI, USA) (Pinheiro-Sousa et al., 2021).

One water sample was collected from each site (A1 and A2) in 2021 and stored in sterile bottles, following the Brazilian Association of Technical Standards (ABNT 9898/1987) guidelines. In the laboratory, trace element concentrations were determined using inductively coupled plasma‒optical emission spectrometry (ICP‒OES; Optima 8300, Perkin Elmer) (Pinheiro-Sousa et al., 2021). The analyzed trace elements included copper (Cu), chromium (Cr), lead (Pb), zinc (Zn), cadmium (Cd), iron (Fe), aluminum (Al), manganese (Mn), mercury (Hg), phosphorus (P), nickel (Ni), arsenic (As), and cyanide (Cn). These elements were selected on the basis of Brazilian environmental regulations (CONAMA 2012), which mandate monitoring in port regions.

### Determination of trace elements in sediment

One sediment sample was collected from each site (A1 and A2) during the *U. cordatus* sampling conducted in 2021. The samples were stored in plastic bags following ABNT 9898/1987 guidelines and sent to the laboratory for chemical analysis of the following trace elements: arsenic (As), cadmium (Cd), lead (Pb), copper (Cu), chromium (Cr), mercury (Hg), nickel (Ni), and zinc (Zn).

The sediment samples were sieved to 200 mesh (74 µm), dried in an oven at 40 °C, ground, and stored in polypropylene containers. Approximately 1.0 g (dry weight) was used for trace element determination after acid digestion. Trace elements were quantified using inductively coupled plasma‒optical emission spectrometry (ICP‒OES Optima 8300, Perkin Elmer) (Pinheiro-Sousa et al., 2021).

For analytical quality control, high-purity analytical grade reagents (PA, Merck) and certified reference materials were used. Solutions were prepared with ultrapure water (Milli-Q Plus, Millipore). All glassware was precleaned by soaking in 5% HNO_3_ (v/v, Merck) for 24 h and rinsing with ultrapure water.

### Biometric analysis

Biometric data from 40 *U. cordatus* specimens, including carapace length (CL), carapace width (CW), cheliped propodus length (CPL), cheliped propodus width (CPW), movable finger length (MFL), movable finger width (MFW) in centimeters, and total body weight (TBW) in grams, were recorded. Measurements were taken using a digital caliper and a precision balance (Jesus et al., 2021).

### Histological analyses of gills and the hepatopancreas

Gill and hepatopancreas 40 samples were dissected using sterile scissors and forceps in the Laboratory of Biomarkers in Aquatic Organisms (LABOAq/UEMA). Subsamples from each organ were collected from every specimen and fixed in Davidson’s solution for 24 h, dehydrated in an ascending ethanol series, cleared in xylene, and embedded in paraffin.

Sections (5 µm thick) were cut and stained with hematoxylin and eosin (H&E) following standard histological procedures (Arockia Vasanthi et al., 2014). Histopathological alterations were examined under a light microscope (Primo Star Zeiss®). Lesions were quantified following the method of Bernet et al. (1999), as adapted by Jesus et al. (2021), and expressed as percentages. In the gills, the total number of lamellae was counted, and lesions were assessed per lamella. For the hepatopancreas, 20 tubules per specimen were analyzed, with all histological lesions recorded.

### Genotoxic analysis

Hemolymph samples (40 specimens) were collected from the movable finger (major dactyl) of each crab using 1 mL hypodermic syringes (21-gauge needles) to minimize hemocyte damage (Nudi et al., 2010). Two smears per specimen were prepared, air dried (20 min), fixed in Canoy’s solution (3:1 methanol: acetic acid, 20 min), and stained with 2% Giemsa in phosphate buffer (pH 8.0, 20 min) (Pinheiro et al., 2013). Micronuclei were counted in 1,000 cells per slide (expressed as MN) using a light microscope (Primo Star Zeiss®) at 1,000× magnification. Samples are considered to be significantly impacted by xenobiotics when the frequency of micronucleated cells exceeds 4 MN% (Scarpato et al., 1990; as adapted for *U. cordatus* by Pinheiro et al., 2013). Nuclear histopathology was scored following Countryman and Heddle (1976).

### Statistical analyses

Abiotic (physicochemical) data from the water samples, biometric measurements, genotoxic responses, and histological lesions in crabs collected in A1 and A2 are expressed as the mean ± standard deviation. The Shapiro–Wilk test was used to assess data normality. Differences between sites (A1 vs. A2) were tested using Student’s *t* test (*p* < 0.05). All the statistical analyses were performed using IBM SPSS Statistics version 20.

## Results and discussion

### Determination of physicochemical parameters and concentrations of trace elements in water

The abiotic parameter results are presented in Table 1. The temperature, pH, and dissolved oxygen levels were within ranges considered suitable for aquatic life according to the Brazilian National Environmental Council (CONAMA Resolution No. 357/2005).

**Table 1:** Abiotic water parameters in water samples from São José Bay (A1) and São Marcos Bay (A2), Maranhão, Brazil, in the rainy and dry seasons of 2021

Parameters	Rainy season		Dry season		
	A1	A2	A1	A2	CONAMA Resolution No. 357/2005 (Brazil)
Temperature	28.59 ± 0.03 ^b,c,d^	29.67 ± 0.04 ^a,b,d^	29.40 ± 0.04 ^a,c^	29.40 ± 0.02 ^a,c^	°C
pH	7.59 ± 0.07	6.89 ± 0.81	7.27 ± 0.92	7.54 ± 0.09	6.5 to 8.5
Dissolved O_2_	5.00 ± 0.26 ^b^	5.22 ± 0.38	5.29 ± 0.02 ^a,d^	5.16 ± 0.09^b^	> 5 mg/L
Salinity	35.3 ± 0.15 ^b,c,d^	19.57 ± 0.71 ^a,b,d^	36.83 ± 0.21 ^a,c,d^	33.73 ± 0.15 ^a,b,c^	Ppm

The values are expressed as the mean ± S.E.M. of the abiotic factors in freshwater

^a^*p* < 0.05, compared with A1 in the rainy season

^b^*p* < 0.05, compared with A1 in the dry season

^c^*p* < 0.05, compared with A2 in the rainy season

^d^*p* < 0.05, compared with A2 in the dry season

Abiotic data collected from São José Bay (A1) and São Marcos Bay (A2) revealed that only dissolved oxygen (in the rainy season) failed to meet Brazilian regulatory legislation (CONAMA Resolution No. 357/2005) for Class III brackish waters (Brazil 2005). Notably, compared with those in A1, dissolved oxygen levels in A2 were significantly lower. Because oxygen is vital for aquatic life, its depletion is often linked to increased organic matter and microbial activity (Fiorucci & Benedetti-Filho, 2005). Fluctuations in dissolved oxygen may result from contaminants originating from agricultural, industrial, or other anthropogenic sources (Castro et al., 2014; Rabitto et al., 2005). The values of other abiotic parameters (salinity, temperature, and pH) were similar in both areas, indicating that A1 was suitable for aquatic life, although the salinity was greater in A1 than in A2. Seasonal rainfall reduces salinity, as freshwater influx is predominant in the rainy season (Carvalho-Neta et al., 2017; Jesus et al., 2020b; Pereira-Filho et al., 2003). Such fluctuations can physiologically stress aquatic organisms (Anger, 2001). pH plays a critical role in contaminant bioavailability, as acidification or alkalinization can alter pollutant uptake by biota (Von Sperling, 2007). Anthropogenic factors, such as domestic and industrial activities, may decrease pH levels. Monserrat et al. (2007) reported that such changes may trigger physiological responses to maintain cellular homeostasis, including osmoregulation (the ability to stabilize extracellular ion concentrations in response to osmotic changes in the environment). While the water temperature is similar in both areas, even minor fluctuations can disrupt nutrient cycling and decomposition rates and affect ecosystem dynamics (Mushore et al., 2024).

Chemical analyses of water revealed high concentrations of trace elements (Table 2), such as Zn, Fe, Al, and Mn (A2 = port region), with levels exceeding the limits established by Brazilian legislation (CONAMA Resolution No. 357 2005).

**Table 2:** Water quality in the regions of São José Bay (A1) and São Marcos Bay (A2) in 2021

Metals	Areas		CONAMA Resolution No. 357/2005 (maximum) (Brasil)
	A1	A2	
Copper (Cu)	< 0.001 mg/L	< 0.001 mg/L	0.005 mg/L
Chromium (Cr)	< 0.010 mg/L	0.050 mg/L	0.05 mg/L
Lead (Pb)	< 0.100 mg/L	< 0.100 mg/L	0.01 mg/L
Zinc (Zn)	< 0.001 mg/L	60 mg/L*	0.09 mg/L
Cadmium (Cd)	< 0.100 mg/L	< 0.100 mg/L	0.005 mg/L
Iron (Fe)	< 1 × 10^–5^ mg/L	0.320 mg/L*	0.3 mg/L
Aluminum (Al)	< 0.100 mg/L	0.430 mg/L*	0.1 mg/L
Manganese (Mn)	< 0.100 mg/L	3.700 mg/L*	0.1 mg/L
Mercury (Hg)	< 2 × 10^–8^ mg/L	< 2 × 10^–8^ mg/L	0.0002 mg/L
Phosphorus (P)	< 1 × 10^–5^ mg/L	2.54 × 10^–3^ mg/L	0.124 mg/L
Nickel (Ni)	< 0.005 mg/L	< 0.005 mg/L	0.025 mg/L
Arsenic (As)	< 0.01 mg/L	< 0.01 mg/L	0.01 mg/L
Cyanide (Cn)	< 1 × 10^–6^ mg/L	< 1 × 10^–6^ mg/L	0.001 mg/L

*Values not in compliance with Brazilian legislation (National Council for the Environment–CONAMA Resolution No. 357/2005)

While certain trace elements (e.g., Mn and Zn) are essential for redox metabolic processes in humans and animals, at elevated concentrations, they become potentially toxic, contributing to soil degradation and water pollution, which ultimately leads to environmental impacts (Minoia et al., 1990; Muszynska & Labudda, 2019). Exposure to these chemical elements in water can increase the susceptibility of aquatic organisms to disease or trigger molecular cellular, physiological, and behavioral alterations (Abba et al., 2018; Biswas & Pramanik, 2016; Jerome et al., 2017; Ragunathan, 2017). Trace elements in water are harmful, toxic, and bioaccumulative in aquatic species, potentially leading to ecological disruptions. Moreover, contaminated organisms may pose health risks to humans upon consumption (Agah et al., 2009; Kadim & Risjani, 2022; Usman et al., 2022).

### Determination of the concentrations of trace elements in sediment

All trace element concentrations in sediments from both study areas (Table 3) were within the limits established by CONAMA Resolution No. 454/2012 (Brasil).

**Table 3:** Trace element concentrations (mg/kg) in sediments from São José Bay (A1) and São Marcos Bay (A2) collected in 2021

Metals	Areas		*Reference values (minimum and maximum)—CONAMA Resolution No. 454/2012 (Brasil)
	A1	A2	
Arsenic (As)	< 0.00001 mg/kg	< 0. 00001 mg/kg	19–70 mg/kg
Cadmium (Cd)	< 0.1 mg/kg	< 0.1 mg/kg	1.2–7.2 mg/kg
Lead (Pb)	< 0.1 mg/kg	< 0.1 mg/kg	46.7–218 mg/kg
Copper (Cu)	12.350 mg/kg	32.565 mg/kg	34–270 mg/kg
Chromium (Cr)	13.7 mg/kg	21.0 mg/kg	81–370 mg/kg
Mercury (Hg)	< 0.0000002 mg/kg	< 0.0000002 mg/kg	0.3–1.0 mg/kg
Nickel (Ni)	< 0.1 mg/kg	< 0.1 mg/kg	20.9–51.6 mg/kg
Zinc (Zn)	16.9 mg/kg	44.53 mg/kg	150–410 mg/kg

*Minimum and maximum values permitted by Brazilian legislation (National Council for the Environment–CONAMA Resolution No. 454/2012)

Sediment analysis of the Amazon coast of the two bays (São José and São Marcos) revealed elevated metal levels (Cu, Cr, and Zn), particularly in A2 (São Marcos Bay). These findings align with those of previous studies reporting higher sediment concentrations of Cd, Cr, Cu, Zn, Ni, and As in São Marcos Bay (Carvalho-Neta et al., 2019; Oliveira et al., 2019). Long-term monitoring along the Maranhão coastline has consistently identified São Marcos Bay (A2) as a hotspot for trace elements in water, sediments, and biota (Castro et al., 2018; Carvalho Neta & Abreu Silva 2010; Oliveira et al., 2019; Pinheiro-Sousa et al., 2013). In addition to water, sediments from port areas (Maranhão, Brazil) exhibit higher concentrations of trace elements (Oliveira et al., 2019). Because sediments have the greatest potential to accumulate trace elements due to very fine silt and clay (Carvalho-Neta et al., 2014). Their toxicity is concerning because of their persistence, bioavailability, bioaccumulation, and biomagnification potential (Chapman, 2008; Ivanina & Sokolova, 2015). Our study further confirmed elevated trace element levels in water exclusively in São Marcos Bay (A2), reinforcing its classification as an impact zone because of industrial and port facilities, which is consistent with prior regional findings (Jesus et al., 2021).

### Biometric analysis

*U. cordatus* specimens from São José Bay (A1) exhibited significantly greater biometric development (*p* < 0.05) than those from São Marcos Bay (A2) in both the rainy and dry seasons (Table 4).

**Table 4:** Comparative biometric data (mean and standard deviation) of *U. cordatus* from São José Bay (A1) and São Marcos Bay (A2), Maranhão, Brazil, in 2021

Parameters	Rainy season		Dry season	
	A1	A2	A1	A2
Total body weight – TBW (g)	164.65 ± 25.09 ^c,d^	109.11 ± 19.74 ^a,b^	163.55 ± 28.34 ^c,d^	130.85 ± 28.05 ^a,b^
Carapace length – CL (cm)	5.63 ± 0.23 ^b,c,d^	4.86 ± 0.38^a^	5.02 ± 0.47 ^a^	4.85 ± 0.35^a^
Carapace width – CW (cm)	7.30 ± 0.42 ^c,d^	6.14 ± 0.84 ^a,b^	7.16 ± 0.44 ^c,d^	6.61 ± 0.46 ^a,b^
Cheliped propodus length – CPL (cm)	6.96 ± 0.61 ^c,d^	5.56 ± 0.75 ^a,b^	6.57 ± 0.94 ^c^	5.91 ± 1.13 ^a^
Cheliped propodus width – CPW (cm)	0.43 ± 0.07^b^	0.41 ± 0.09 ^b^	0.48 ± 0.11 ^a,c,d^	0.42 ± 0.09 ^b^
Movable finger length – MFL (cm)	3.88 ± 0.64 ^c,d^	3.12 ± 0.53 ^a,b^	3.85 ± 0.64 ^c,d^	3.17 ± 0.68 ^a,b^
Movable finger width – MFW (cm)	0.87 ± 0.13 ^c,d^	0.78 ± 0.06^a,b^	0.89 ± 0.14 ^c,d^	0.76 ± 0.08 ^a,b^

The values are expressed as the mean ± standard deviation of *U. cordatus* biometric data (*n* = 20)

^*a*^*p* < 0.05, compared with A1 in the rainy season

^*b*^*p* < 0.05, compared with A1 in the dry season

^*c*^*p* < 0.05, compared with A2 in the rainy season

^*d*^*p* < 0.05, compared with A2 in the dry season

Biometric data revealed that all morphometric parameters of *U. cordatus* collected from site A2 (São Marcos Bay) were lower than those recorded for individuals from site A1 (São José Bay). This pattern aligns with previous findings in areas impacted by residential and port activities (Jesus et al., 2020a, 2021; Oliveira et al., 2019) and likely reflects energy reallocation from growth to the detoxification process in contaminated environments (Moureaux et al., 2011; Oliveira et al., 2019; Santos et al., 2016).

### Histological analysis of gills and the hepatopancreas

Histological examination of *U. cordatus* gills revealed distinct pathological patterns between the minimally impacted Facão Island (A1) and the port-affected Coqueiro (A2) in the rainy and dry seasons (Table 5). Crabs from A1 predominantly exhibited cuticle detachment and hemocytic nodules (indicative of parasitic infection), whereas those from A2 showed more severe architectural damage, including marginal canal deformation, lamellar collapse, pillar cell rupture, and necrosis, suggesting significant environmental stress at the impact site.

**Table 5:** Quantitative analysis (mean ± standard deviation) of the occurrence of histological lesions in the gills of *Ucides cordatus* collected in the rainy and dry seasons in São José Bay (A1) and São Marcos Bay (A2), Maranhão, Brazil, in 2021

Types of histological lesions	Rainy season		Dry season	
	A1	A2	A1	A2
Marginal canal deformation	0.267 ± 0.056 ^c,d^	0.495 ± 0.055 ^a,b,d^	0.291 ± 0.041 ^c,d^	0.320 ± 0.034 ^a,b,c^
Cuticle detachment	0.134 ± 0.039 ^b,c,d^	0.045 ± 0.011 ^a,d^	0.055 ± 0.013 ^a,d^	0.015 ± 0.007 ^a,b,c^
Lamellar collapse	0.308 ± 0.089 ^b,d^	0.361 ± 0.084 ^b,d^	0.065 ± 0.035 ^a,c^	0.104 ± 0.049 ^a,c^
Hyperplasia	0.002 ± 0.001 ^b,c^	0.014 ± 0.005 ^a,b,d^	0.000 ± 0.000 ^a,c,d^	0.004 ± 0.002 ^b,c^
Rupture of pillar cells	0.532 ± 0.081 ^b^	0.567 ± 0.086 ^b^	0.393 ± 0.057 ^a,c^	0.398 ± 0.07 1^c^
Hemocytic nodule (parasites)	0.007 ± 0.003 ^d^	0.005 ± 0.003	0.005 ± 0.002	0.002 ± 0.001 ^a^
Cuticle rupture	0.098 ± 0.039 ^b,c^	0.020 ± 0.014 ^a,d^	0.042 ± 0.011 ^a,d^	0.077 ± 0.016 ^b,c^
Lamellar collapse with swelling	0.000 ± 0.000 ^c^	0.011 ± 0.007 ^a,b,d^	0.000 ± 0.000 ^c^	0.000 ± 0.000 ^c^
Primary lamella narrowing	0.006 ± 0.002 ^c,d^	0.021 ± 0.001 ^a,b,d^	0.006 ± 0.001 ^c,d^	0.009 ± 0.001 ^a,b,c^
Necrosis	0.120 ± 0.026 ^b,c,d^	0.237 ± 0.081 ^a,b^	0.013 ± 0.009 ^a,c,d^	0.308 ± 0.083 ^a,b^

The values are expressed as the mean ± standard deviation of the histological alterations found in the gills of *U. cordatus* in the rainy and dry seasons (*n* = 20)

^a^*p* < 0.05, compared with A1 in the rainy season

^b^*p* < 0.05, compared with A1 in the dry season

^c^*p* < 0.05, compared with A2 in the rainy season

^d^*p* < 0.05, compared with A2 in the dry season

Histological analysis of gill tissues revealed lesions in *U. cordatus* from both sites, with a higher prevalence in A2 specimens. Such alterations are common histopathological responses to sublethal pollutant exposure and serve as a defensive mechanism against environmental stress (Negro, 2015). The severity of these alterations highlights the increasing ecological pressure in the region in recent years, as such lesions indicate the loss of cellular and tissue integrity (Negro & Collins, 2017). Because the gills serve as the primary interface for osmoregulation, ionic balance, excretion, and xenobiotic filtration, gill damage in *U. cordatus* signals significant physiological disruption (Arman, 2021; Oliveira et al., 2019).

The gill lesions in *U. cordatus* represent defense mechanisms against environmental contaminants (Winkaler et al., 2001). In crustaceans, contaminant absorption occurs primarily through membranes, gills, and ingestion (Lezcano et al., 2015; Pinheiro & Toledo, 2010; Rainbow, 2007). Gill lesions occur more rapidly and indicate acute contamination (Carvalho-Neta et al., 2017). Prolonged exposure to polluted environments induces adverse effects, including organ deformation and tissue bioaccumulation (Winkaler et al., 2001).

Histopathological analysis of the *U. cordatus* hepatopancreas revealed distinct seasonal alterations across the sites (Table 6). Crabs collected in A1 primarily exhibited vacuolated B cells, pyknotic nuclei, and luminal abnormalities, whereas A2 crabs showed more severe rainy-season lesions, including epithelial delamination, vacuolated B cells, and necrosis, which transitioned to dry-season manifestations of luminal distortion, pyknotic nuclei, and myoepithelial damage, demonstrating progressive tissue degeneration at the impacted site.

**Table 6:** Quantitative analysis (mean ± standard deviation) of hepatopancreatic lesions in *U. cordatus* in the rainy and dry seasons in São José Bay (A1) and São Marcos Bay (A2), Maranhão, Brazil, in 2021

Types of histological lesions	Rainy season		Dry season	
	A1	A2	A1	A2
Abnormal lumen	19.500 ± 1.395 ^b^	18.300 ± 4.426 ^b^	15.259 ± 3.945 ^a,c,d^	19.600 ± 1.353 ^b^
Pyknotic nuclei	8.150 ± 1.269 ^b,d^	5.400 ± 1.486 ^d^	5.600 ± 0.955 ^a,d^	11.800 ± 1.200 ^a,b,c^
Epithelial delamination	1.000 ± 0.316 ^c,d^	6.950 ± 1.468 ^a,b,d^	1.250 ± 0.403 ^c,d^	3.900 ± 0.656 ^a,b,c^
Parasite	0.250 ± 0.099^c^	0.450 ± 0.169 ^a,b,d^	0.150 ± 0.081 ^c^	0.250 ± 0.096 ^c^
Vacuolated B cell	14.250 ± 0.839 ^b,c,d^	9.200 ± 1.631 ^a,d^	9.900 ± 1.613 ^a,d^	5.996 ± 1.340 ^a,b,c^
Damaged myoepithelial layer	11.650 ± 1.236 ^d^	11.800 ± 1.475 ^d^	11.850 ± 0.892 ^d^	16.650 ± 0.567 ^a,b,c^
Hemocytic infiltration	0.300 ± 0.127 ^b^	0.150 ± 0.082 ^b^	0.000 ± 0.000 ^a,c,d^	0.150 ± 0.109 ^b^
Necrosis	2,000 ± 0.091 ^b,c,d^	12.950 ± 1.685 ^a,b,d^	0.150 ± 0.081 ^a,c,d^	4.100 ± 0.092 ^a,b,c^

The values are expressed as the mean ± standard deviation of the number of histological lesions in the hepatopancreas of *U. cordatus* (*n* = 20)

^a^*p* < 0.05, compared with A1 in the rainy season

^b^*p* < 0.05, compared with A1 in the dry season

^c^*p* < 0.05, compared with A2 in the rainy season

^d^*p* < 0.05, compared with A2 in the dry season

The hepatopancreas, the main detoxification organ in crustaceans, plays a critical role in mitigating toxicant effects in the circulatory system (Kang et al., 2012). Lesions in the hepatopancreas indicate chronic contamination (Carvalho-Neta et al., 2017). There are four cell types: E cells (embryonic cells that give rise to other cell types), R cells (absorptive or reabsorptive), F cells (fibrillar cells responsible for the secretion of digestive enzymes), and B cells (which are involved in intracellular digestion and the excretion of enzymes and xenobiotics, often appearing as vesicle-like structures) (Chavez-Crooker et al., 2001; Kang et al., 2012; Schuwerack & Lewis, 2003; Zilli et al., 2003).

Among the alterations observed in the hepatopancreas of the crabs analyzed in the present study, abnormal lumen, damaged myoepithelial layer, deformation, and vacuolated B cells were the most prominent in the specimens from both bays. Star-shaped lumen deformity arises from morphological changes in tubular epithelial cells (Maharajan et al., 2015), whereas abnormal lumens reflect excretory and detoxification stresses (Bhavan & Geraldine, 2000; Suryavanshi et al., 2009). Vacuolated B cells release holocrine secretions under contaminant-induced stress, exacerbating necrosis, tubule delamination, and lumen abnormalities (Negro, 2015).

### Genotoxic analysis

The results of the genotoxic analysis of *U. cordatus* hemolymph assessed through micronucleus testing in the rainy and dry seasons are presented in Table 7. The hemolymph of the analyzed crabs revealed that the frequency of lesions in the port-impacted area (A2) was greater than that in the A1 specimens.

**Table 7:** Mean ± standard deviation of hemolymph analysis in *U. cordatus* collected from São José Bay (A1) and São Marcos Bay (A2), Maranhão, Brazil, in 2021

Parameters	Rainy season		Dry season	
	A1	A2	A1	A2
Micronucleated cell	0.100 ± 0.010 ^b,c,d^	0.550 ± 0.029 ^a,b,d^	0.250 ± 0.012 ^a,c,d^	0.150 ± 0.008 ^a,b,c^
Binucleated cell	0.300 ± 0.012 ^b,c,d^	0.100 ± 0.068 ^a,b,d^	0.000 ± 0.000 ^a,c^	0.000 ± 0.000 ^a,c^
Cell in karyorrhexis	2.350 ± 0.055 ^b,c,d^	5.050 ± 0.185 ^a,b,d^	0.300 ± 0.018 ^a,c^	0.300 ± 0.016 ^a,c^
Nuclear invagination	0.000 ± 0.000 ^c^	1.631 ± 0.036 ^a,b,d^	0.000 ± 0.000 c	0.000 ± 0.000 ^c^
Pyknotic cell	3.900 ± 0.075 ^b,c,d^	7.450 ± 0.169 ^a,b,d^	1.850 ± 0.050 ^a,c,d^	1.600 ± 0.051 ^a,b,c^
Budding cell	0.000 ± 0.000 ^c^	0.050 ± 0.005	0.000 ± 0.000 ^c^	0.000 ± 0.000 ^c^
Cell in karyolysis	2.350 ± 0.047 ^b,c,d^	3.900 ± 0.128 ^a,b,d^	0.100 ± 0.068 ^a,c,d^	0.000 ± 0.000 ^a,b,c^

The values are expressed as the mean ± standard deviation of hemolymph analysis in *U. cordatus* (*n* = 20)

^a^*p* < 0.05, compared with A1 in the rainy season

^b^*p* < 0.05, compared with A1 in the dry season

^c^*p* < 0.05, compared with A2 in the rainy season

^d^*p* < 0.05, compared with A2 in the dry season

These data indicate that *U. cordatus* exposed to xenobiotic elements may experience genotoxic effects, including chromosomal integrity impairment (Venturoti et al., 2019). Such exposure can disrupt chromosomal migration during mitosis, leading to micronucleated cells or other nuclear abnormalities in erythrocytes (Calado et al., 2020). Notably, the mean numbers of micronuclei and nuclear alterations, particularly pyknotic cells, were greatest in the crabs from A2 (São Marcos Bay). The presence of pyknotic cells indicates nuclear and chromatin condensation responses, in which the cell degenerates in the final stages and eventually undergoes fragmentation; this process often culminates in karyorrhexis, a marker of nonprogrammed cell death (Aki et al., 2023).

Micronuclei formation reflects exposure to aneugenic or clastogenic compounds, with micronuclei being smaller than the main cell nuclei (D’Costa et al., 2019). These structures arise from whole chromosomal fragments that fail to reach the poles during cell division because of the absence of centromeres, thereby causing cytokinesis disruption (Heddle et al., 1991; Vincent-Hubert et al., 2011). Disrupted cell renewal kinetics further exacerbate this type of cytogenetic damage (Dixon et al., 2002; Vincent-Hubert et al., 2011). As sensitivity biomarkers of genotoxicity, micronuclei and nuclear abnormalities effectively reveal xenobiotic-induced damage in aquatic organisms (Calado et al., 2020; Falfushynska et al., 2021; Oyeniran et al., 2021).

This study confirms the convergence between environmental chemical analyses and biological responses in *U. cordatus*, reinforcing the value of biomarkers as early warning indicators of ecological stress in anthropogenically impacted estuaries. To advance monitoring and management efforts, future research should prioritize (1) integrated programs combining biomarker assessments in sentinel species with systematic chemical analyses of water and sediments to resolve spatiotemporal contamination dynamics, (2) comparative studies across aquatic species to evaluate biomarker sensitivity and identify optimal bioindicators, and (3) expanded biomarker panels targeting diverse physiological pathways to improve the detection of sublethal and chronic effects. By consolidating these approaches, the combined use of biomarkers and chemical data can refine environmental diagnostics and support evidence-based conservation of vulnerable estuarine ecosystems.

## Conclusions

The crab *Ucides cordatus* is considered to be an effective biomonitor for histological and genotoxic biomarkers, revealing organismal exposure to elevated trace element levels in water and sediments across the studied areas. By integrating physicochemical data with biomarker analyses in *U. cordatus*, this study established a robust framework for environmental assessment in impacted coastal zones.

Notably, the higher frequencies of necrosis, micronuclei formation, and pyknotic cell alterations observed in crabs collected from the port region (A2) clearly reflect the contamination history of this area, as further evidenced by elevated metal concentrations in both the water and sediments. These findings highlight that the biomarkers employed respond not only to the presence of contaminants but also possibly to their bioaccumulation and chronic toxic effects resulting from continuous exposure to trace elements, reinforcing their potential as scientific tools for environmental monitoring.

The convergence of chemical and biological data further highlights the ability of sentinel organisms, such as *U. cordatus,* to deliver early and integrated warnings related to the impacts of environments with multiple stressors, which is particularly relevant in areas subject to intense port and industrial activities. Collectively, the biometric, histopathological and genotoxic alterations emphasize the value of using multilevel analytical biomarker approaches to comprehensively evaluate ecological degradation and inform management strategies in vulnerable bays.

## Data Availability

No datasets were generated or analysed during the current study.
